# Decision region analysis for generalizability of artificial intelligence models: estimating model generalizability in the case of cross-reactivity and population shift

**DOI:** 10.1117/1.JMI.11.1.014501

**Published:** 2024-01-25

**Authors:** Alexis Burgon, Berkman Sahiner, Nicholas Petrick, Gene Pennello, Kenny H. Cha, Ravi K. Samala

**Affiliations:** U.S. Food and Drug Administration, Center for Devices and Radiological Health, Office of Science and Engineering Laboratories, Division of Imaging, Diagnostics, and Software Reliability, Silver Spring, Maryland, United States

**Keywords:** generalizability, decision region, represented and unrepresented subgroups, vicinal distribution, cross-reactivity, population shift

## Abstract

**Purpose:**

Understanding an artificial intelligence (AI) model’s ability to generalize to its target population is critical to ensuring the safe and effective usage of AI in medical devices. A traditional generalizability assessment relies on the availability of large, diverse datasets, which are difficult to obtain in many medical imaging applications. We present an approach for enhanced generalizability assessment by examining the decision space beyond the available testing data distribution.

**Approach:**

Vicinal distributions of virtual samples are generated by interpolating between triplets of test images. The generated virtual samples leverage the characteristics already in the test set, increasing the sample diversity while remaining close to the AI model’s data manifold. We demonstrate the generalizability assessment approach on the non-clinical tasks of classifying patient sex, race, COVID status, and age group from chest x-rays.

**Results:**

Decision region composition analysis for generalizability indicated that a disproportionately large portion of the decision space belonged to a single “preferred” class for each task, despite comparable performance on the evaluation dataset. Evaluation using cross-reactivity and population shift strategies indicated a tendency to overpredict samples as belonging to the preferred class (e.g., COVID negative) for patients whose subgroup was not represented in the model development data.

**Conclusions:**

An analysis of an AI model’s decision space has the potential to provide insight into model generalizability. Our approach uses the analysis of composition of the decision space to obtain an improved assessment of model generalizability in the case of limited test data.

## 1 Introduction

Understanding the limitations of artificial intelligence (AI) models in medical devices is crucial to ensuring that they continue to be used in a safe and effective manner. An important real-world safety and/or effectiveness issue is when a model does not generalize well to the intended target population or users. However, determining the limitations of a model’s generalizability based only on a single independent test dataset is not trivial. Estimated generalizability is typically evaluated using an external test dataset sequestered from multiple sources^1^^,^^2^ while accounting for and mitigating any sources of bias.^3^ This test set is created with the assumption that it is a good approximation of the intended target user or patient population of the medical device. In the case of medical imaging devices, intended populations can change over time, and large and diverse test datasets are difficult to obtain,^4^ making it challenging to provide timely access to AI technologies in health care while ensuring a proper level of safety and effectiveness. This is a dilemma considering the potential advantages that AI technologies^5^ have to offer in improving health care delivery. The need for methods of assessing model generalizability is becoming even more important with the advent of evolving algorithms that may be piece-wise modified by the device developer,^6^ potentially updated continuously^7^ or tailored to a local population^8^^–^^10^ or specific subgroups.^11^ Thus, there is a need to innovate and help overcome some of the generalizability assessment limitations in AI-enabled medical devices.

There are a variety of approaches that can be used, either independently or in conjunction with each other, to assess a model’s ability to generalize. One such approach is the use of explainable AI techniques. An explainable model provides users with information about how its decision was reached, which can help indicate when models are being used in situations in which they cannot generalize well and the model does not have sufficient knowledge to make a meaningful prediction.^12^ Explainable AI approaches seek to increase the transparency of how deep convolutional neural networks (DCNNs) arrive at their outputs. However, model transparency is ill-defined^13^ and claims of explainability are difficult to validate. Another approach for ensuring that models are only used on populations for which their predictions are reliable is to use open-set or anomaly detection methods to determine when a sample is outside of the intended target population.^14^ Although these approaches have the potential to increase the safety of AI-enabled devices, the situations in which they can be realistically applied are limited. These approaches require fitting an additional classifier or rely on access to extensive knowledge of the training data distribution. Furthermore, a diverse test dataset is required to evaluate the effectiveness of open-set and anomaly detection approaches. Out-of-distribution detection can also be accomplished using Bayesian models due to their inherent capabilities at both capturing and using the data distributions for prediction.^15^ Despite these capabilities, Bayesian models have limited practical applicability as they are computationally expensive, training a robust model is difficult, and they have been shown to perform worse than deep ensembles in the case of data shift.^16^

Current methods that can reliably evaluate model generalizability require the availability of large, diverse datasets, which may be an unrealistic expectation in the field of medical imaging. An analysis of the model’s decision function and accompanying decision space has the potential to provide information on how it will perform on data beyond those available and how it can be applied to any model, regardless of the architecture.^17^ In this work, we present a novel approach for a test-time assessment of AI model generalizability with limited data that leverages the existing diversity in the data to create virtual samples for each class, which are used to obtain a deeper understanding of the model’s decision space (Sec. 2). Using the non-clinical task of classifying a patient subgroup from chest x-rays (CXR) as an example (Sec. 3), we demonstrate that decision region composition analysis can provide some additional understanding of model generalizability beyond what can be gleaned from the performance on the original limited available data (Sec. 4). Our work, along with parallel efforts in the areas of open-set, anomaly detection, and explainable AI methods, may help in assessing the safety of AI-enabled medical devices in an efficient manner.

## 2 Decision Region Analysis

Analysis of an AI model’s decision space involves mapping a change in the input data to a change in the output class to determine the way in which altering different input features affects the model’s prediction. For simple classifiers with low-dimensional inputs, the decision space can be analyzed relatively easily using the dimensions of the input as the axes to create a visualization to show the effect of each input feature on the model’s prediction. The increased dimensionality and complexity of DCNNs make decision space analysis difficult^18^ as it requires a way to create a meaningful representation of a high-dimensional space.

One approach to creating an interpretable representation of the decision space is to use manifold learning. Manifold learning involves the use of dimensionality reduction techniques to create a low dimensional representation of high dimensional data that maintains the intrinsic structure.^19^ Dimensionality reduction techniques can provide useful information on the relative similarity of samples from a model perspective. However, dimensional reduction techniques are always subject to the distortion of angles and distances,^20^ and they have varying degrees of subjectivity as the projection depends heavily on the specific parameters and dataset used. Dimensionality reduction techniques do not provide information about a model’s generalizability as they cannot provide information about the decision space beyond the available data without the use of an inverse projection.^20^ Analysis of the decision space without the use of dimensionality reduction requires the creation of virtual samples.^17^ Creating virtual samples increases the density of samples in the decision space and allows for the assessment of the decision space beyond the available samples. Because of the limited information that dimensionality reduction techniques provide regarding the decision space beyond the available data, virtual sample generation is used to characterize the decision space instead.

There are several ways to create virtual samples and not all of them provide the same insight into the decision space. Section 2.1 introduces properties of the decision space for DCNNs related to the model’s generalizability and the characterization thereof. The approach used in this study to generate virtual samples is presented in Sec. 2.2, and the assessment of the generalizability of a DCNN via characterization of the decision space is given in Sec. 2.3.

### 2.1 Structure of the Decision Space

An understanding of the decision space is needed to ensure that the virtual samples generated are useful for generalizability assessment. This relationship is the foundation for our proposed approach of decision region analysis for generalizability (DRAGen). The decision space of DCNNs becomes structured as the model trains, developing decision boundaries around a manifold formed by the set of samples provided during training.^21^ The resulting decision space is highly structured near the data manifold and lacks structure elsewhere. This results in the model being unable to generalize well on samples that fall outside of the structured region of the decision space surrounding the data manifold.^21^ Thus, to assess the model generalizability using the decision space, we must determine not the structure of the data manifold but rather how far beyond the data manifold this structure extends. However, quantifying the distance in the decision space is an ongoing challenge^22^ due to its high dimensionality. Due to the difficulty in measuring distance in the decision space, multiple samples that are already known to lie near the manifold are used to generate virtual samples. Although the finite test set may not sufficiently represent the entire intended population, it does represent a portion of it. Therefore, the data manifold is known to reside within the portion of the decision space representing the intended population. Using multiple original samples during the generation of virtual samples ensures that the virtual samples lie near the manifold as each original sample serves to anchor the distribution of virtual samples to the region of the decision space near the manifold. This approach has been used to characterize the decision boundary using adversarial examples.^23^ However, this approach by itself is restricted to the available samples rather than the behavior of the decision space beyond the available data. Thus, increasing the diversity of the sample space through the generation of virtual samples can help determine the extent of the structure beyond the data manifold.

### 2.2 Vicinal Distribution Generation

**Fig. 1:**
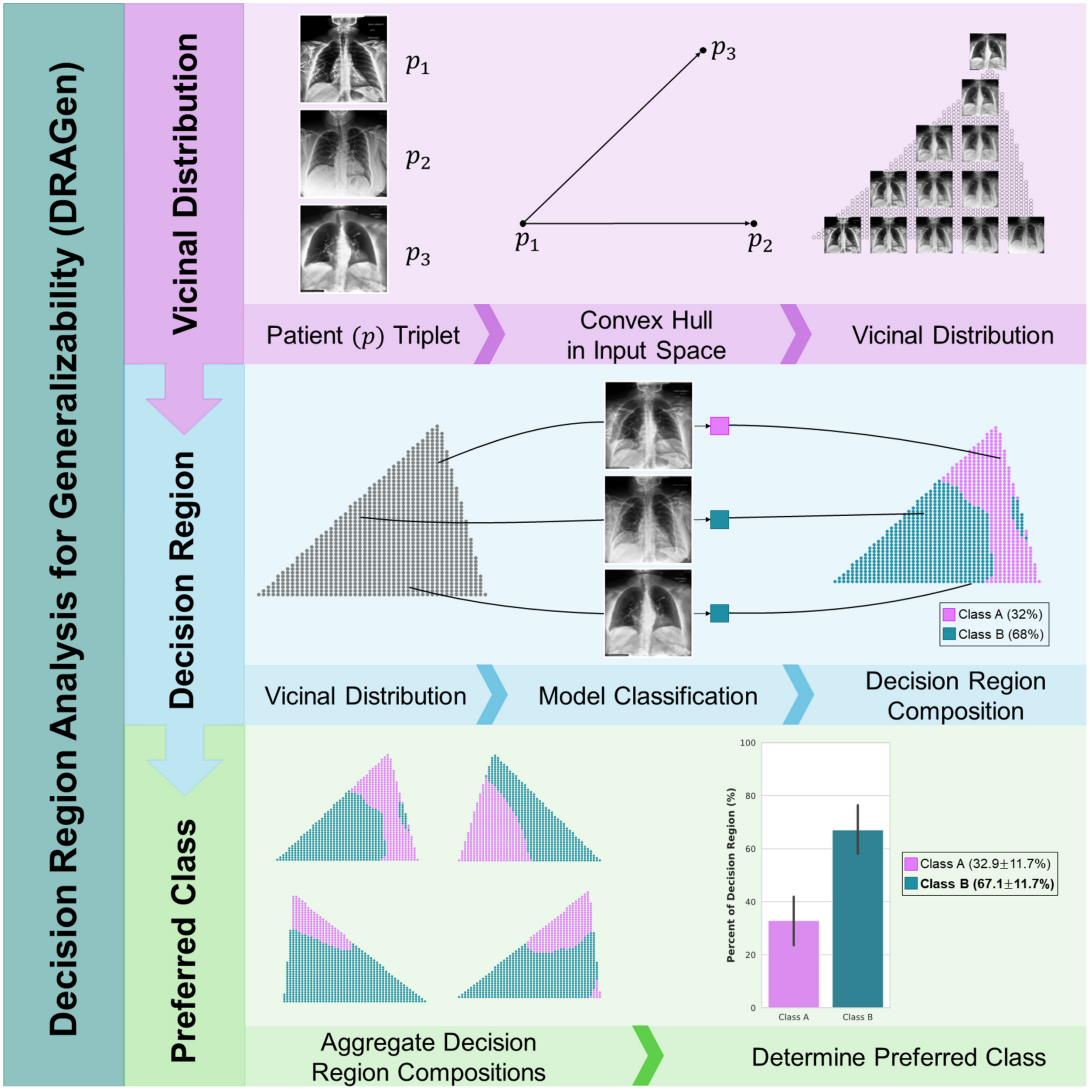
Overview of the proposed approach to characterize the model’s decision regions (Sec. 2). A vicinal distribution of virtual samples is created using linear interpolation along the plane between a triplet of three samples in the input space (Sec. 2.2). Model classification of the virtual samples allows for the mapping of the model output to the input space and visualization of a region of the decision space. Aggregation of the composition of decision regions from a multitude of triplets provides insight into the model’s behavior on samples beyond the available dataset (Sec. 2.3).

Linear interpolation presents a simple and robust way to generate a vicinal distribution of virtual samples from the original data distribution as was done in the mixup method.^24^ The linear interpolation of two samples has been shown to be sufficient for increasing the model generalizability when used during training,^24^ and to characterize the properties of decision boundaries when used at test-time evaluation.^23^ However, by interpolating between a “triplet” of three images sampled from the original dataset, the virtual samples are generated closer to the existing data manifold where the decision boundaries of the model are structured.^21^ The triplet of samples is composed of three selected points in the input space that can be used to construct a plane spanning the triplet in the input space.^21^ Virtual samples are created by uniformly sampling the constructed plane. Model inference on the virtual samples allows for a region of the decision space to be visualized by mapping the model classification to a two-dimensional plane spanned by the triplet in the input space. This allows for the characterization of the region of the decision space that lies in the vicinity of the triplet samples, thus providing additional anchoring locations to the region of the decision space near the manifold. The process is shown in Fig. 1. The use of vicinal distributions for the analysis of the decision region composition was explored in a preliminary work^25^ that evaluated the decision region for a classifier of patient sex and image acquisition method from CXR. This study builds upon that preliminary work to connect the composition of the decision region to the model’s generalizability.

### 2.3 Decision Region Composition

Model classification of the vicinal distribution of virtual samples provides information on how the model will behave when presented with samples that are not represented by the model development data but still fall near the structured regions of the decision space. However, the virtual samples lack associated labels and attribute information. Previous work using linearly interpolated virtual samples during model training demonstrates increased generalizability when the labels are linearly interpolated along with the sample images.^24^ Nevertheless, the same approach cannot be applied to virtual samples generated for model evaluation as there is no way to determine the “true class” of a virtually created image. To overcome this limitation, the decision regions in this study are generated from triplets in which the samples within a triplet all belong to the same class. Each decision region’s composition is then evaluated with respect to the class of the triplet rather than the model’s performance on individual virtual samples.

As explained in Sec. 2.1, the difficulty in measuring the distance in an interpretable way complicates the analysis of the decision space and makes it challenging to directly measure the area of the decision space dedicated to each class. To overcome this limitation, in our approach the composition of the regions of the decision space is estimated using the percentage of vicinal distribution samples classified as belonging to each class. As the virtual samples of the vicinal distribution are evenly distributed between the samples of the input triplet, they can be used as a proxy to the area of that region of the decision space dedicated to that class.

## 3 Materials and Methods

The AI models developed in this study were trained to classify CXR images into patient attributes: sex, race, age group, and COVID status. Although these tasks are not all clinically relevant, this systematic experimental setup is designed to develop and validate the enhanced generalizability assessment approach that can be used for any AI-enabled medical imaging classification task, as described in Sec. 2.

### 3.1 Data

Data from the Medical Imaging and Data Resource Center (MIDRC) Open-A1 repository were used in this study, and the associated patient subgroups are provided in Appendix A. Patients with missing label information for one or more of the classification tasks (sex, race, age, or COVID status) were not included in the study as they could introduce noise into the training data and would be impossible to evaluate. A single image with the imaging study date closest to the patient’s polymerase chain reaction (PCR) test date was selected for each patient. Random image selection was used in the event that a patient did not have a recorded PCR test or to make a selection between multiple images taken with the same number of days from a PCR test. The images used in this study were restricted to anterior-posterior and posterior-anterior view positions to prevent unintended bias. Additionally, due to an imbalance between the number of patients with computed radiography (CR) and digital radiography (DX) imaging studies, only CR imaging studies were included. All images were converted from 16-bit DICOM to 8-bit JPEG file format. Images were downsampled using bilinear interpolation such that their shortest dimension was 320 pixels, followed by a center crop, resulting in all images being 320×320  pixels.^21^

#### 3.1.1 Represented and unrepresented subgroups

Patients were divided into distinct subgroups as defined by the combination of the patient attributes of sex, race, COVID status, and age group (e.g., female-black-negative-40s). A limited dataset was simulated by restricting the data used during model development to sixteen patient subgroups. The patient attributes represented in the model development data are shown in Table 1. All subgroups that were not used during model development are considered unrepresented in this study and withheld for use during the evaluation (Sec. 3.4) of our proposed generalizability assessment method. Additional extended trials using patient subgroups defined only by patient sex, race, and COVID status are included in Appendix D.

**Table 1:** Represented and unrepresented patient groups selected in this study. For each attribute, the two groups with the most patients were represented in the model development data. All other groups are considered unrepresented and withheld for evaluation of the generalizability assessment.

Patient attribute	Represented groups	Unrepresented groups
Sex	Female	None
	Male	
Race	Black or African American	American Indian or Alaska Native
	White	Asian
		Native Hawaiian or Other Pacific Islander
COVID status	Negative	None
	Positive	
Age group	40 to 49	<40
	60 to 69	50 to 59
		70+

#### 3.1.2 Data partitions

**Fig. 2:**
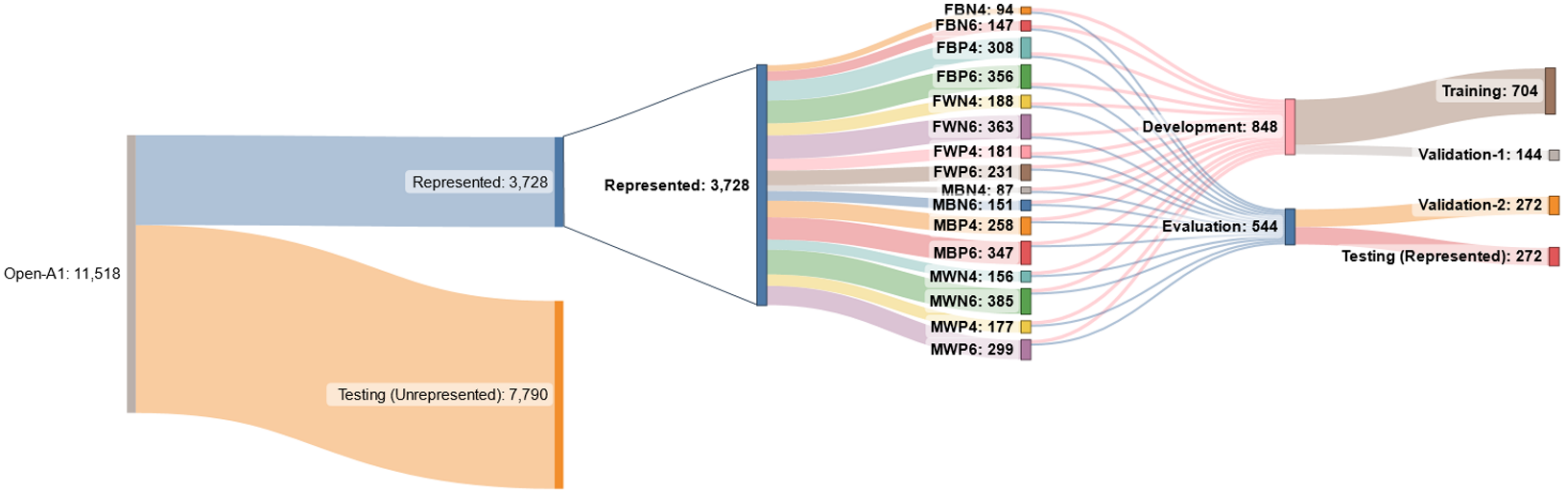
Data partition subgroup information. Patients are separated into represented or unrepresented, depending on subgroup (Sec. 3.1.1). Patient subgroups for represented patients are defined by the combination of the patient attributes of sex [female (F) or male (M)], race [black (B) or white (W)], COVID status [positive (P) or negative (N)], and age group [40s (4) or 60s (6)]. Unrepresented patients are withheld for evaluation of the proposed generalizability assessment (Sec. 3.4). Due to the selection of a single image per patient, the numbers shown represent both the number of patients and images included in the study.

The represented subgroups were divided into four partitions (training, validation-1, validation-2, and testing), each stratified by patient such that each subgroup was equally represented in each partition, as shown in Fig. 2. 50% of the data were used for model training, 10% for model selection and determination of thresholds (validation-1), 20% for decision region generation and performance evaluation (validation-2), and 20% for independent testing and verification of the validation-2 performance. The represented patient subgroups were separated into model development and evaluation data. This separation was repeated five times, resulting in five separate model development and evaluation sets. The model development data were then randomly divided into training and validation-1 partitions. The training/validation-1 partitioning process was repeated five times for each model development set. This resulted in 25 different training and validation-1 sets, 5 sets for each of the evaluation sets. The 25 different combinations were used for sensitivity analysis of variations due to both development and evaluation data.

### 3.2 Model Development

The models used in this study are ensembles of 20 individual ResNet-18 models. The ensembles were created by training 20 models on the same training data, each with a different random state. The output score for each sample was obtained by averaging the output scores of all models in the ensemble. This ensemble approach was used to produce more consistent decision region composition estimations by reducing the effects of epistemic uncertainty.^26^ The decision space created by the ResNet-18 architecture has been previously explored^21^ and was found to be relatively consistent between different random initializations when compared with other modern architectures, such as DenseNet-121. Each model in the ensemble was trained until the model converged to a loss of <0.2 and a standard deviation of ≤0.05 across five sequential epochs.

The models used in this study were pretrained using contrastive self-supervised learning (CSL), which has been shown to perform better than ImageNet pretraining for CXR classification.^27^ Specifically, an approach called MoCo-CXR^28^ was used; it is an adaptation of the momentum contrast (MoCo)^29^ approach for use with CXR data. MoCo maximizes the agreement between positive pairs of images (an image and augmentations thereof) while minimizing the agreement between negative pairs (any other pair of images). This allows for the learning of visual representations without class labels. Following CSL pretraining, an additional dense layer was added to the models. This pretrained model was used for all simulations performed in this study.

Output score thresholds were determined separately for each classification task by selecting the operating point at which the false positive rate (FPR) and false negative rate (FNR) were approximately equal between the binary outputs in the validation-1 set. This ensured that imbalances in the decision region composition were not due to the threshold favoring prediction of certain classes.

### 3.3 Decision Region Generation

All decision regions used for analysis were generated using a triplet of patient images that were randomly selected from patients who belonged to the same subgroup, as defined by the combination of patient COVID status, sex, race, and age group. This ensured that there was a proper reference class for every task and reduced the likelihood that the virtual samples contained unrealistic combinations of patient characteristics. Fifty sample triplets were used for each of the sixteen represented patient subgroups. The composition of the decision region was determined for each sample triplet. The decision region compositions were then aggregated by determining the average decision region composition for each patient subgroup, producing a single estimate of the composition of the decision space surrounding each patient subgroup. Increasing the number of triplets did not have a substantial effect on the measured decision region compositions beyond fifty triplets (Appendix C).

### 3.4 Evaluation

As described in Sec. 3.1, the patient subgroups used during model development were limited to sixteen subgroups, and the remaining subgroups were withheld for the evaluation of the decision region composition-based assessment of model generalizability. The limited number of represented patient subgroups simulates a situation in which the data available for model development is an imperfect representation of the intended population. As the four patient attributes used to denote subgroup are also the attributes by which the model classifies samples, two different methods of evaluation are utilized. The evaluation methods, cross-reactivity (Sec. 3.4.1) and population shift (Sec. 3.4.2), are distinguished by whether the class of the sample is unrepresented with respect to a specific classification task. The type of evaluation used for each task and the attribute(s) by which a sample is considered unrepresented are shown in Table 2 and described in the following sections.

**Table 2:** Evaluation approach used depending on the specific classification task and the patient attribute(s) by which a patient is considered unrepresented.

Classification task	Unrepresented patient attribute(s)		
	Race	Race and age group	Age group
Sex	Population shift		
Race	Cross-reactivity		Population shift
COVID status	Population shift		
Age group	Population shift	Cross-reactivity	

#### 3.4.1 Cross-reactivity

Cross-reactivity occurs when two classes appear similar to the classification model.^30^ The particular form of cross-reactivity considered in this study is when a sample whose class is not under consideration for classification, i.e., a patient who is unrepresented with respect to the specific classification task, is classified into one of the classes under consideration. For example, as the model development data did not contain any patients who are 70 or older, a patient who is 70 years old would be considered a case of cross-reactivity for the classification of patient age group. Furthermore, the model could not make a correct age group classification for this patient as the only two potential outputs for age group classification are 40s and 60s. Cross-reactivity does not directly consider how well the model generalizes to its intended population but rather the way in which the model will behave when used beyond its intended scope. Due to the similar presentation of different diseases, it is likely that AI-enabled medical devices will be used for patients who fall outside the device’s intended use scope. For patient race and age group classification tasks, there are classes that were not represented in the training and validation data or in the potential model classification outputs. For these tasks, the models can be evaluated in the context of cross-reactivity, in which the model must make a classification despite the model output classes not including the correct class.

#### 3.4.2 Population shift

Population shift refers to a situation in which the patient is represented with respect to the classification task in question but has other unrepresented attributes. For example, as the model development data contained female patients but did not contain patients who were 70 or older, a female patient who is 70 years old would be considered a case of a population shift in regards to the classification of patient sex but a case of cross-reactivity for the classification of patient age group. This method of evaluation provides insight into how well the decision region-based assessment of generalizability captures the behavior of the model when the data used during model development lacked the diversity of the intended population. For the classification of patient sex and COVID status, all classes present in the dataset were represented in the training and validation data. Therefore, all unrepresented patient subgroups are considered part of a population shift for the classification of patient sex and COVID status. A population shift also occurs for race and age classification, when a patient belongs to an unrepresented subgroup but the attribute that is being classified was represented in the model development data.

## 4 Results

### 4.1 Decision Region-Based Assessment of Generalizability

**Fig. 3:**
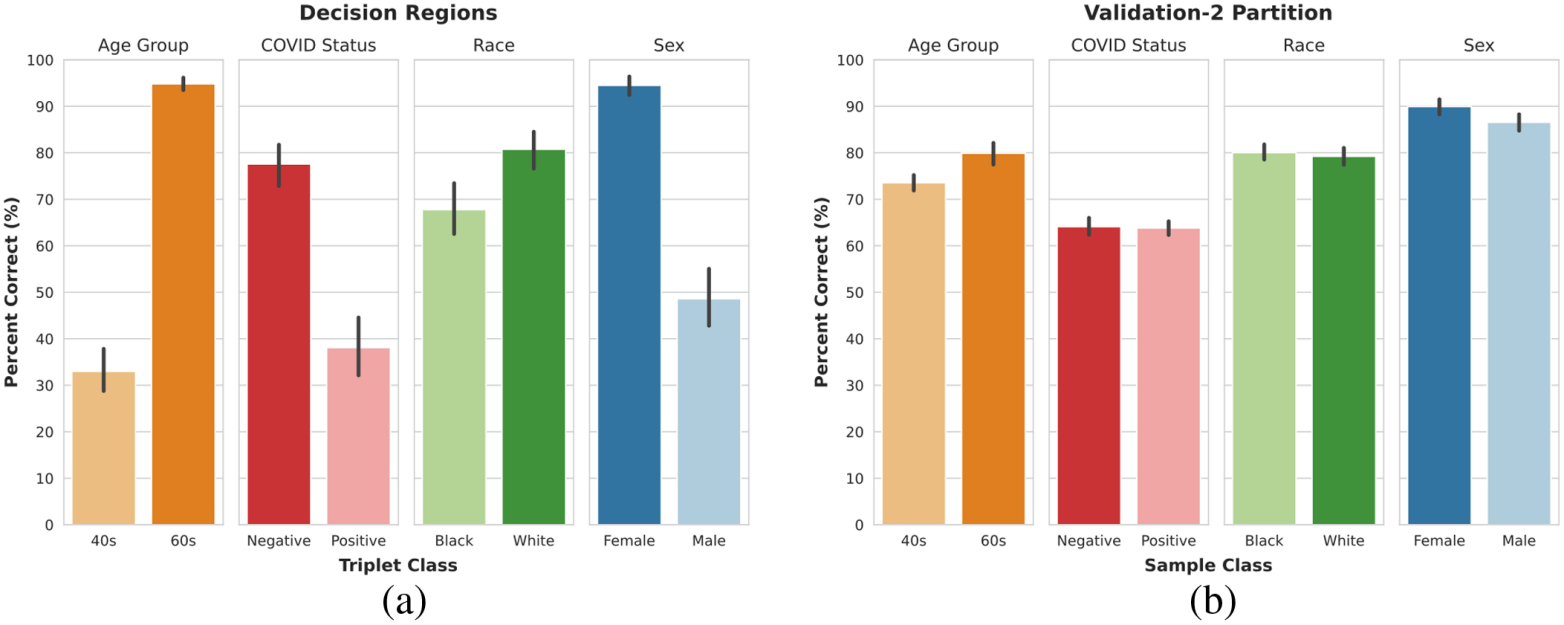
(a) Portion of the decision region classified as belonging to the same class as the input triplet, separated by the classification task. (b) Portion of samples in the validation-2 subset that were correctly identified, separated by classification task.

The composition of the decision regions was aggregated for each class to obtain the average portion of the decision space belonging to the same class as the sample triplet. Each classification task shows a disproportionately large portion of the decision space belonging to one preferred output class despite comparable performance on the original samples of the validation-2 partition from which the triplets were sampled, as shown in Fig. 3. The preferred classes are 60s (patient age group), negative (patient COVID status), white (patient race) and female (patient sex).

### 4.2 Performance under Population Shift

**Fig. 4:**
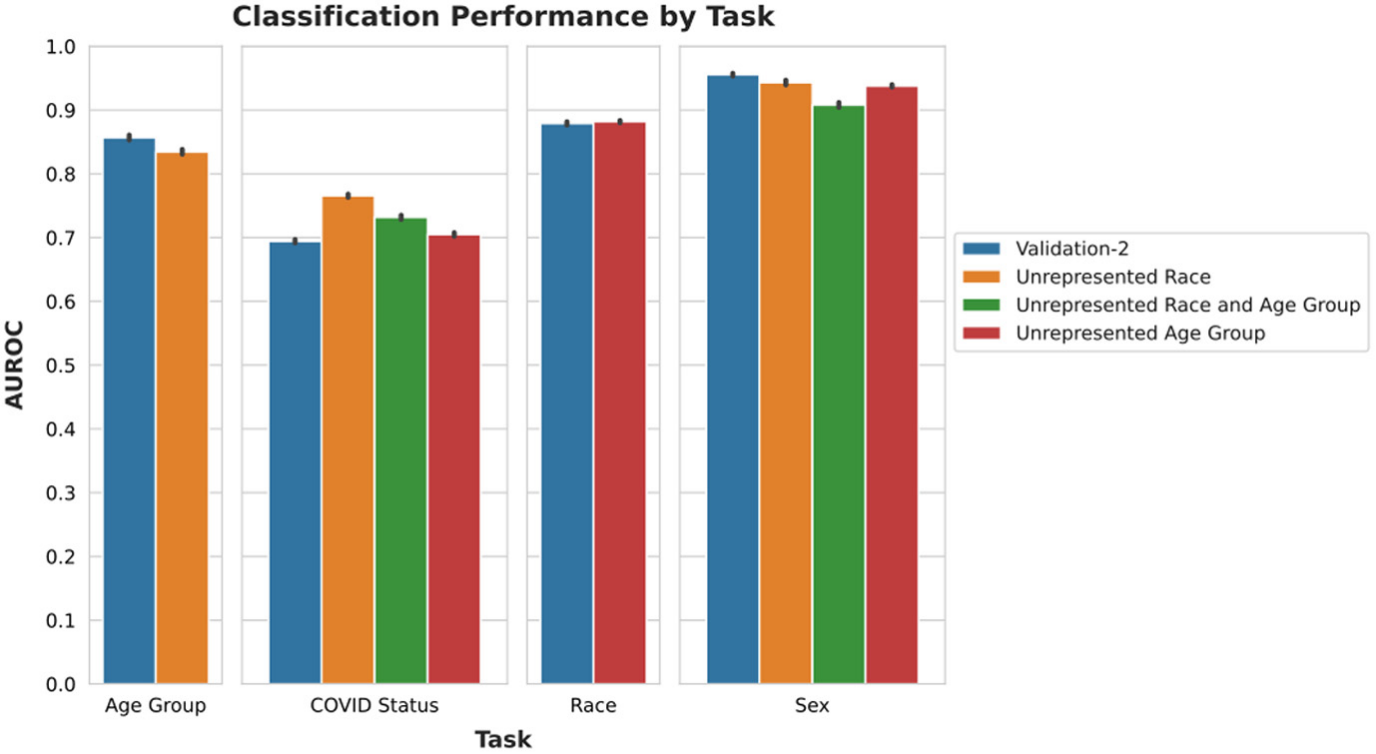
Classification performance by task on the validation-2 dataset and unrepresented subgroups.

**Fig. 5:**
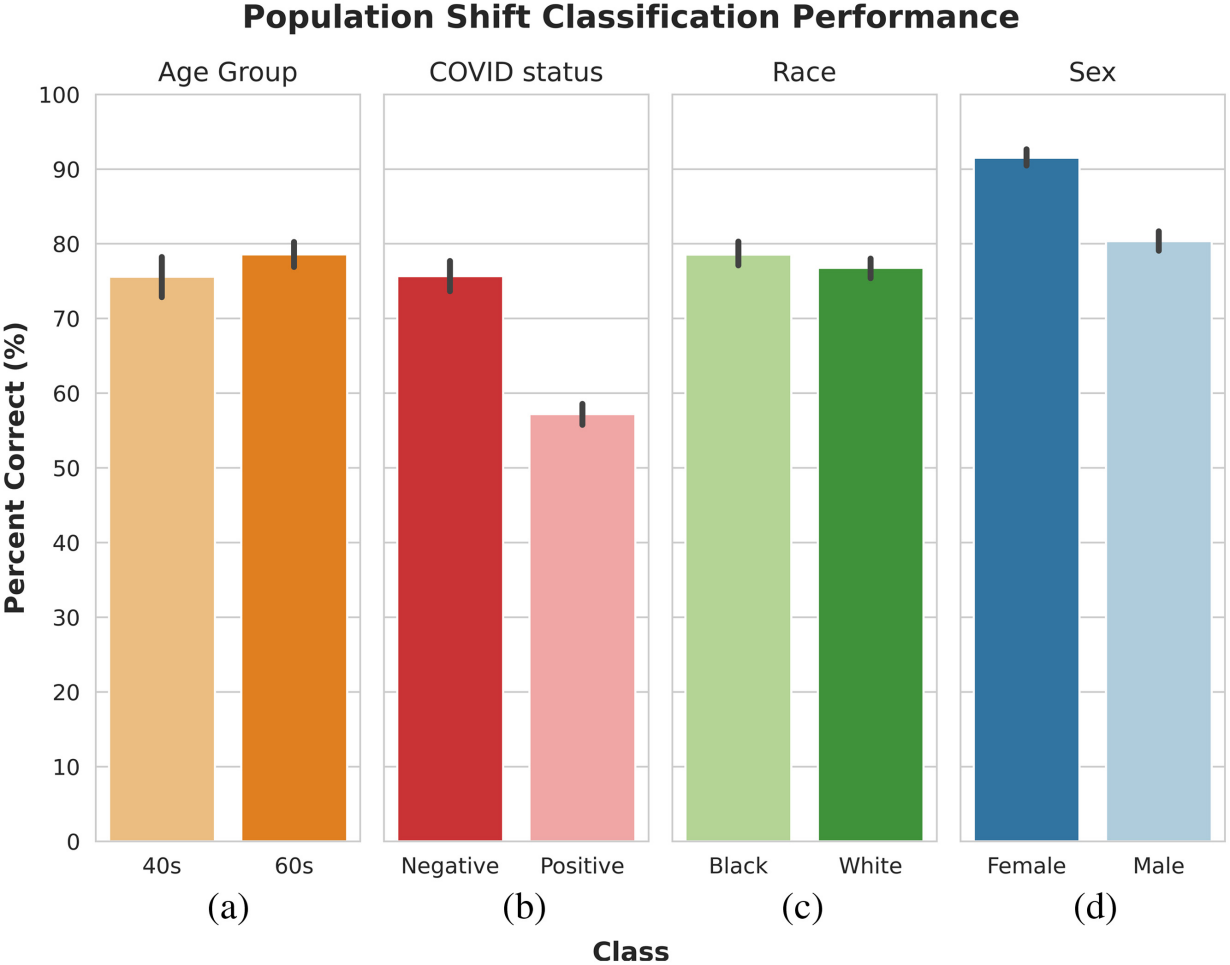
(a)–(d) Population shift classification performance. The percentage of unrepresented patients correctly classified with respect to each classification task. For example, for the COVID detection task, the population shift in the “negative” column represents the percentage of COVID negative patients that were correctly identified in the unrepresented set shown in Fig. 2. Please refer to the “unrepresented groups” column in Table 1.

The overall area under the receiver operating characteristic curve (AUROC) did not decrease substantially on the unrepresented subgroups for each task (Fig. 4). However, the false positive rates of each class are no longer comparable (Fig. 5). This shows that, even though the model’s performance did not decrease, the type of mistakes changed, with the model being much more likely to make an incorrect classification on the non-preferred class than on the preferred class. The tendency to overpredict the model’s preferred class in the event of a population shift is stronger for the classification of patient COVID status and sex than for patient race or age group, as seen in Fig. 5.

### 4.3 Cross-reactivity Performance

**Fig. 6:**
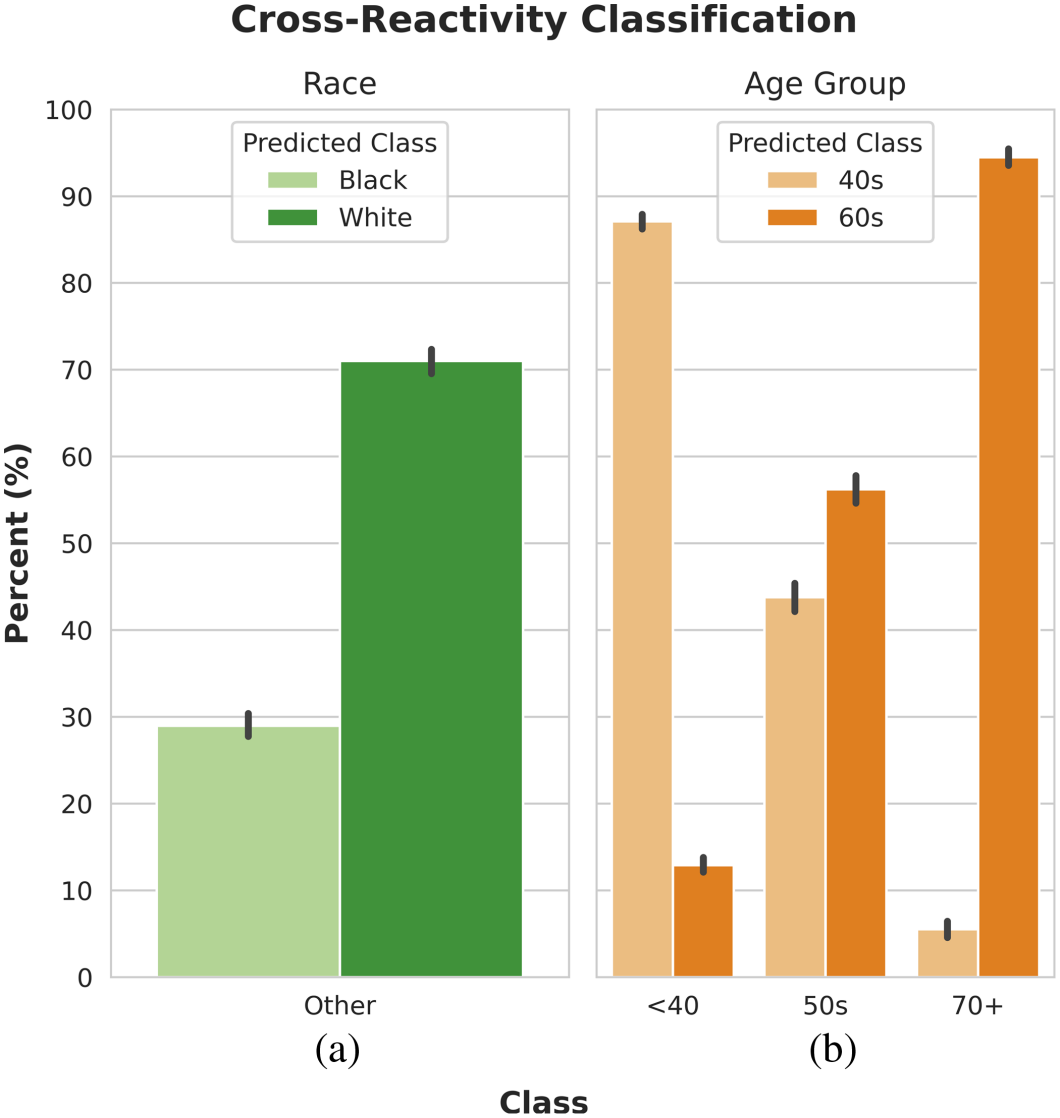
(a) and (b) Cross-reactivity classification performance. The percentage of unrepresented patients classified as each represented class.

The model’s race classification behavior shows the cross-reactivity performance when presented with patients whose race was represented in neither the model development data nor the available classification outputs. Figure 6(a) shows that the model has an increased tendency to classify patients as the preferred race classification: white.

Evaluating the model’s patient age group classification in the event of cross-reactivity reveals that patients under 40 and 70 or older are more likely to be classified as being in their 40s and 60s, respectively [Fig. 5(b)]. However, there is still a slight tendency to over-predict the preferred class (60s), with more patients under 40 being classified as in their 60s than patients 70 or older being classified as in their 40s. Additionally, patients whose age is between the represented subgroups, i.e., patients who are in their 50s, are more likely to be classified as being in their 60s than in their 40s.

## 5 Discussion and Conclusion

In this study, we focus on a generalizability assessment from the perspective of assessing scenarios in which the model may likely fail. Such scenarios are unpredictable and difficult to detect; however, understanding how the model is likely to behave is important to ensuring safe usage. Our proposed approach of the decision region composition analysis provides an enhanced assessment of model generalizability by leveraging the available variability in the data to predict how the model will likely behave on unrepresented subgroups. The decision region analysis for assessing generalizability in this study consistently indicated that a disproportionately large portion of the decision space belongs to a single preferred class for each classification task. These preferred classes could not be inferred from the performance on the validation-2 dataset (Fig. 3) or the test dataset for represented subgroups (Appendix B), necessitating the use of vicinal distributions of virtual samples.

The generated virtual samples do not represent realistic patients themselves; however, they are created using combinations of characteristics already present in the available data, allowing for increased sample diversity compared with the finite available evaluation set. In this study, the emphasis is on assessing the model’s ability to generalize to its intended population, rather than creating deliberately adversarial samples or observing how the model behaves on samples far beyond its intended scope, which should be detected by quality control. The results indicate each classification task had one preferred class despite comparable performances across classes on the evaluation (validation-2) dataset. This was also confirmed on the test set, the results of which are shown in Appendix B. Furthermore, the models showed a tendency to overpredict samples as belonging to the preferred class when presented with data that was not represented in the model development dataset.

In evaluating the model performance under a population shift (Sec. 3.4.2), the model showed a tendency to overpredict samples as belonging to the preferred classes for certain tasks and not others. The classification tasks of patient race and age group only experienced a population shift with respect to one patient attribute (age attribute for race classification task and race attribute for age classification task). For these tasks, the tendency to prefer one class was minor or nonexistent. As such, the model was not more likely to correctly classify the age of patients in their 60s (78.6%±4.3%) than patients in their 40s (75.6%±7.1%) under a distribution shift in their race. Additionally, the model was not substantially more likely to correctly classify the race of patients who were either black (78.6%±4.0%) or white (76.8%±3.7%) under a distribution shift in their age. For the classification of patient sex and COVID status, however, the model experienced a population shift with respect to up to two attributes. Consequently, the model was more likely to correctly classify the sex of patients who were female (91.5%±4.7%) than those who were male (80.4%±5.5%). A similar trend is observed for the classification of patient COVID status, which was more likely to correctly classify patients belonging to the preferred class of negative (75.7%±9.1%) than patients belonging to the positive class (57.2%±6.1%). This may indicate that, for our dataset, the model can continue to generalize well if only one of the patient attributes is unrepresented but begins to struggle when more than one patient attribute is unrepresented.

As with a population shift, in the case of cross-reactivity, the model overpredicts samples as belonging to preferred classes. 71.0%±5.0% of the patients whose race was not represented in the model development data were classified as white. This behavior was predicted by the decision region analysis, which indicated that white was the preferred class for classification of patient race. For age group classification, unlike the classification of patient race, there are inherent connections between the represented and unrepresented. It can be assumed that patients would be most similar to the represented age group closest to their true age. Despite this inherent connection, the model displayed a slight tendency to overpredict the preferred class. 12.9%±3.2% of patients under the age of 40 were classified as 60 to 69, and only 5.5%±3.5% of patients older than 70 were classified as 40 to 49. Furthermore, 56.2%±5.9% of the patients whose true age was between the represented age groups were classified as 60 to 69.

Together, the population shift and cross-reactivity simulate the different non-optimal scenarios in which we can reasonably expect that the model may be used. The results of the decision region analysis can help us predict the model’s behavior in such scenarios, even when such behavior is not evident from a performance assessment of the original data.

The observed preferred classes were consistent across different data partition combinations (Sec. 3.1.2), and the preferred classes for patient sex, race, and COVID status were the same in the extended trials (Appendix D). As mentioned in Sec. 3.2, the output score thresholds were determined separately for each class based on the threshold at which the FPR and FNR were equal for the validation-1 dataset. This ensured that the preferred class behavior observed in the decision region analysis was not the result of the score threshold favoring a specific class.

Although the presence of the preferred class behavior is shown, it is not clear what factors impact which class becomes the model’s preferred class. The preferred classes for patient sex, race, age group and COVID status were consistent across different repetitions of random data partitioning. This suggests that the preferred class is heavily impacted by one or more of the experimental components that were constant throughout all of our experiments: model architecture, data repository, and training method. This study was performed using a single model architecture and data from a single repository; however, the approach is applicable to any image classification model and more work is needed to understand the effects of model architecture and dataset on the model’s selection of the preferred class. The preferred class may be affected by the model pretraining or utilization of different training approaches, such as active learning, few-shot learning, or semi-supervised learning. In a prior study^31^ pretraining between CXR data and ImageNet was compared, and it was found that CXR pretraining results in an improved and more robust performance. This suggests that there is a difference in decision space composition due to a difference in pretraining techniques. Additional studies are required to understand the degree to which pretraining affects a model’s decision space. Our approach uses a finite test set to infer how the model is likely to behave on a larger, more diverse population. However, we do not currently have a way to determine how large the finite test set must be to provide useful information. Considering that the virtual samples used for decision space characterization are created using the samples in the finite test set, the virtual sample diversity is dependent on the diversity in the original test set. The quality of the decision space characterization is also affected by the number of triplets generated from the test set. This was explored in Appendix C, where it is shown that the composition of the decision region did not change substantially as a result of increasing the number of triplets per subgroup above fifty. This number is likely affected by the existing diversity in the finite test set and does not explore the effects of the evaluation set size itself but the number of triplets generated from the evaluation set. Additionally, the virtual samples are evaluated with respect to the class of the triplet samples from which they are generated, following the assumption that the characteristics of the virtual samples are most similar to the characteristics associated with the triplet’s subgroup. The virtual sample characteristics should be explored further to ensure that they remain most similar to the subgroup characteristics of the source triplet. Furthermore, in this work, data diversity regarding only patient subgroups was considered; it did not consider diversity relating to other factors that can impact model generalizability, such as institutional or image variability. As mentioned in Sec. 3.1, only patients with CR imaging studies were included. Although the differences between CR and DX imaging studies are a substantial source of image variability, considering image acquisition as an additional attribute for the determination of patient subgroups would have decreased the size of our data such that there would be fewer than three patients per subgroup in the validation-2 dataset, making the selection of triplets impossible. As image acquisition could not feasibly be considered while determining the patient subgroup, using both imaging acquisition methods could have introduced bias. Thus, to avoid bias due to image acquisition, DX imaging studies were not used. Although our current approach can indicate how a model is likely to behave when used on data for which it cannot generalize, we do not currently have a method of determining the extent to which a model can generalize from the decision region analysis. In other words, we can say that a model is likely to overpredict a certain class when presented with patients for whom it cannot generalize, but we cannot make any quantitative assessment on the models’ relative generalizability by comparing the decision region analysis of two different models. The extent of a model’s generalizability can theoretically be assessed from the decision space by determining how far the decision boundaries extend beyond the manifold (as mentioned in Sec. 2.1). However, this assessment would rely on having accurate measures of the distance in the decision space as well as a method to determine how far beyond the manifold the intended population extends.

Although previous methods of characterizing the decision space have investigated the geometric and topological properties of the decision space^18^ or attempted to characterize the model’s robustness to adversarial examples based on the distance between natural images and decision boundaries,^32^ this is, to our knowledge, the first work connecting the analysis of the decision space to model generalizability to other medical images, rather than in the context of robustness to adversarial examples. A robust assessment of model generalizability is essential to ensuring the safe and effective use of AI in medical devices. With the perpetual limited availability of large datasets in medical imaging, it is important to utilize the available data to the fullest extent possible. To this end, our approach leverages the limited diversity of the available data to provide additional information about model generalizability, and it can be performed during traditional performance assessment. An enhanced generalizability assessment of the model through decision region analysis may allow for a more accurate evaluation of the model safety and effectiveness prior to clinical implementation.

## 6 Appendix A: Patient Subgroup Information

As described in 3.1, a limited number of patient subgroups were used during model development to simulate an extremely limited dataset. The subgroups that were represented in the model development data were determined by the number of available patients in each subgroup. Table 3 shows the number of patients per subgroup as defined by patient sex, race, COVID status, and age group. Of the 188 available patient subgroups, only forty contain fifty or more patients. The represented patient races were limited to black and white as they were the only patient races with fifty or more patients in each of the age groups. Patient age was similarly restricted by selecting the age groups with the largest number of patients.

**Table 3:** Demographics of the patients included in the Open-A1 repository. Only patients with sex, race, age, and COVID status attribute information and CR imaging studies are included.

COVID status	Sex	Race	Age group					
			<40	40 to 49	50 to 59	60 to 69	70+	Total
Negative	Female	American Indian or Alaska Native	0	1	1	1	1	4
		Asian	7	10	10	21	16	64
		Black or African American	152	94	140	147	146	679
		Native Hawaiian or Other Pacific Islander	1	0	1	0	1	3
		White	278	188	308	363	778	1915
	Male	American Indian or Alaska Native	0	0	1	1	1	3
		Asian	17	10	9	11	16	63
		Black or African American	170	87	146	151	99	653
		Native Hawaiian or Other Pacific Islander	1	0	0	1	0	2
		White	279	156	331	385	692	1843
Positive	Female	American Indian or Alaska Native	4	3	1	2	4	14
		Asian	11	14	12	10	12	59
		Black or African American	503	308	405	356	333	1905
		Native Hawaiian or Other Pacific Islander	2	1	0	1	0	4
		White	213	181	237	231	358	1220
	Male	American Indian or Alaska Native	2	1	1	2	0	6
		Asian	15	13	14	16	21	79
		Black or African American	435	258	387	347	253	1680
		Native Hawaiian or Other Pacific Islander	1	2	1	1	0	5
		White	249	177	237	299	351	1313

## 7 Appendix B: Test Partition Classification Performance

**Fig. 7:**
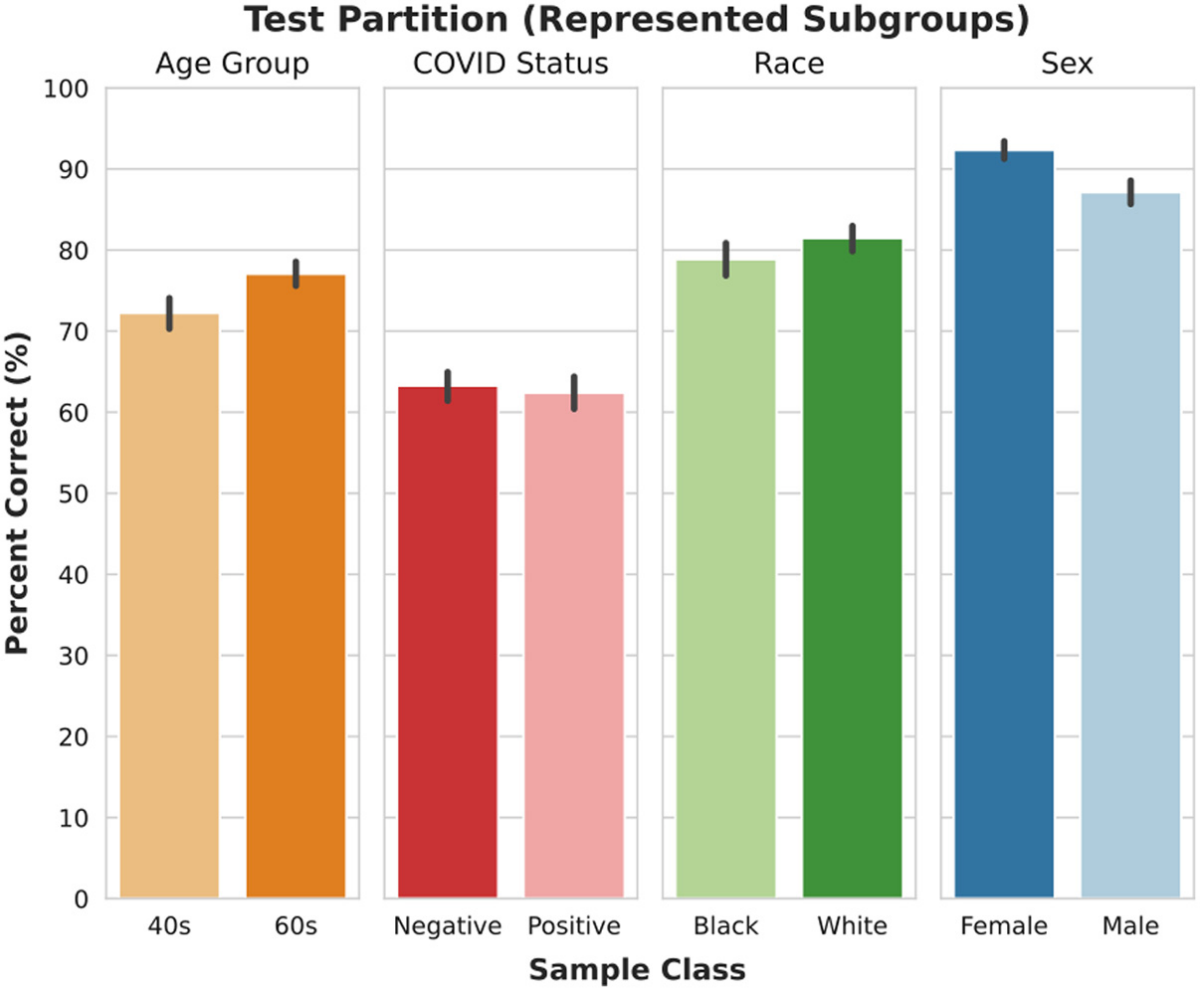
Percent of patients correctly classified in the test partition.

As introduced in Sec. 3.1.2, 20% of the patients belonging to the represented subgroups were withheld for further evaluation in the form of the test partition. The model classification performance on this partition can be seen in Fig. 7. For each class, the percent of samples that were correctly classified is comparable to the model performance on the validation-2 set, as shown in Table 4.

**Table 4:** Percent of patients correctly classified in the test and validation-2 partitions, divided by task and class.

Task	Class	Percent correct (test)	Percent correct (validation-2)
Sex	Female	92.3(±2.9)	89.9(±4.3)
	Male	87.1(±4.0)	86.6(±4.5)
Race	Black	78.9(±5.3)	80.0(±4.3)
	White	81.5(±4.1)	79.2(±4.8)
COVID status	Negative	63.3(±4.5)	64.1(±4.8)
	Positive	62.4(±5.2)	63.8(±4.0)
Age group	40–49	72.1(±4.8)	73.6(±4.2)
	60–69	77.1(±4.1)	79.9(±5.9)

## 8 Appendix C: Effect of Number of Triplets on Decision Region Composition

**Fig. 8:**
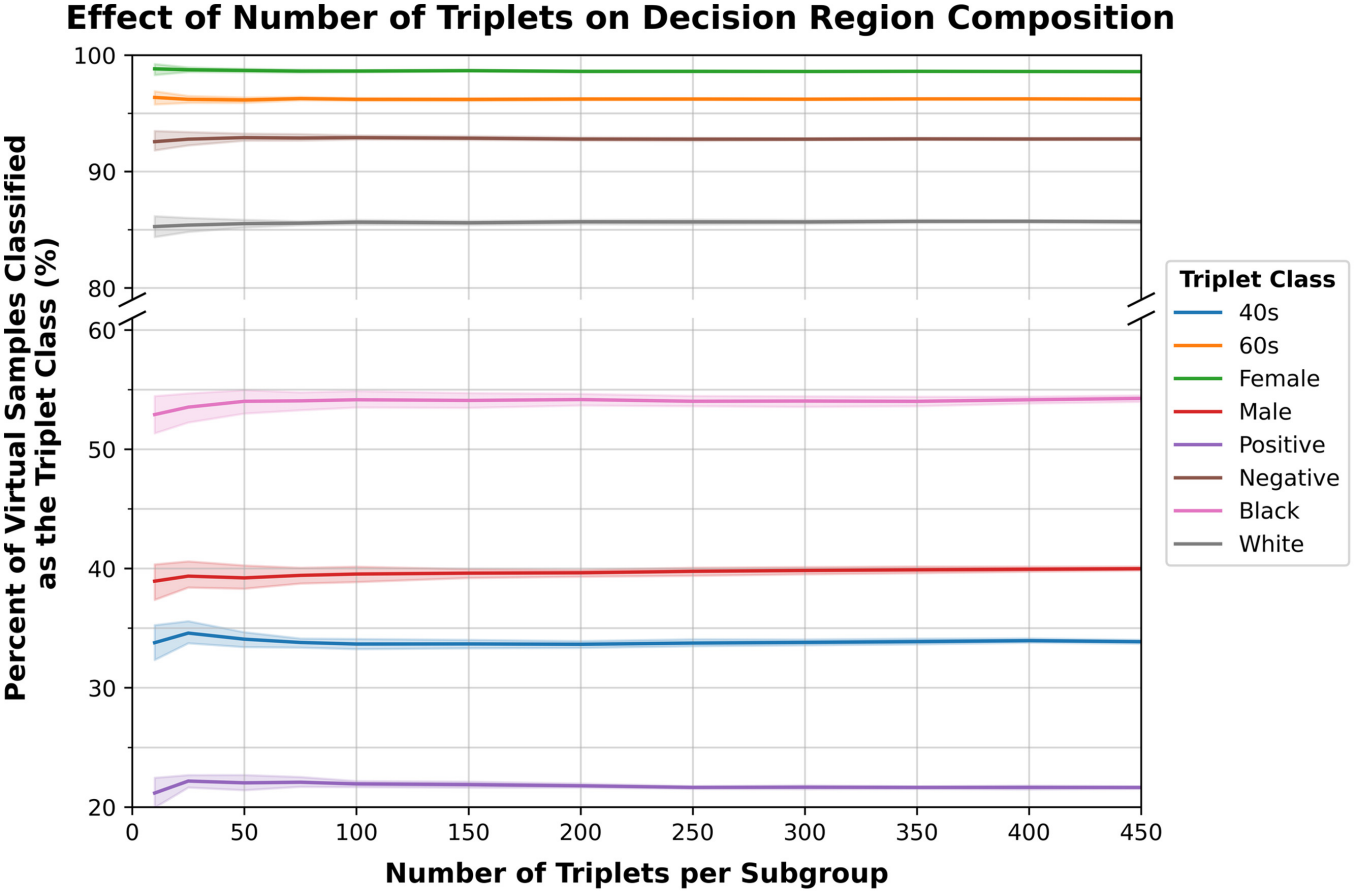
Effect of the number of triplets on the decision region composition. The composition of the decision region is consistent provided at least fifty triplets are selected per subgroup. Repeated random sampling of each number of triplets per subgroup provides the standard deviation.

The preferred classes in this study were determined using the aggregation of the compositions of decision regions created from triplets of images selected at the test time. Fifty triplets were selected per patient subgroup. Additional triplets were generated for one of the ensembles to ensure that this number of triplets was sufficient for properly characterizing the composition of the decision space surrounding the available samples. The additional generated triplets were randomly sampled and aggregated to simulate the use of different numbers of triplets per subgroup. As shown in Fig. 8, for the dataset used in this study, the number of triplets does not have a significant effect on the determined composition of the decision regions, provided at least fifty triplets are selected per patient subgroup.

## 9 Appendix D: Effect of Size of the Model Development Data to Identify the Preferred Class

To determine the effect of the size of the data used during model development on the applicability of the decision region-based assessment of generalizability, an additional experiment was performed using a larger dataset during model development. The same Open-A1 data were used with fewer subgroups with larger sizes. In this experiment, the patient attributes used for both subgroup determination and model classification tasks were limited to sex, race, and COVID status. This resulted in a larger represented portion of the data.

**Fig. 9:**
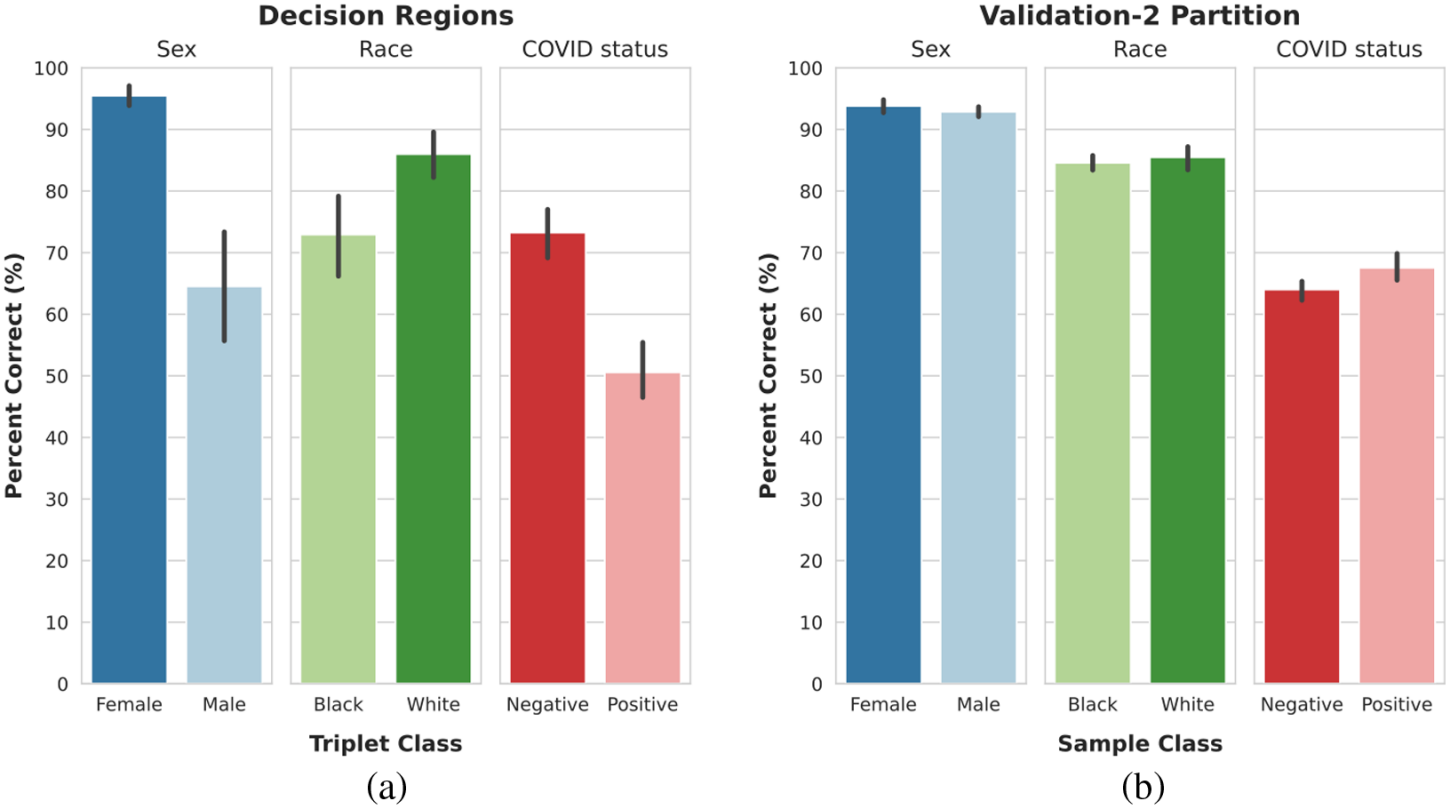
(a) Portion of the decision region classified as belonging to the same class as the input triplet, separated by classification task. (b) Portion of samples in the validation-2 subset that were correctly identified, separated by classification task.

**Fig. 10:**
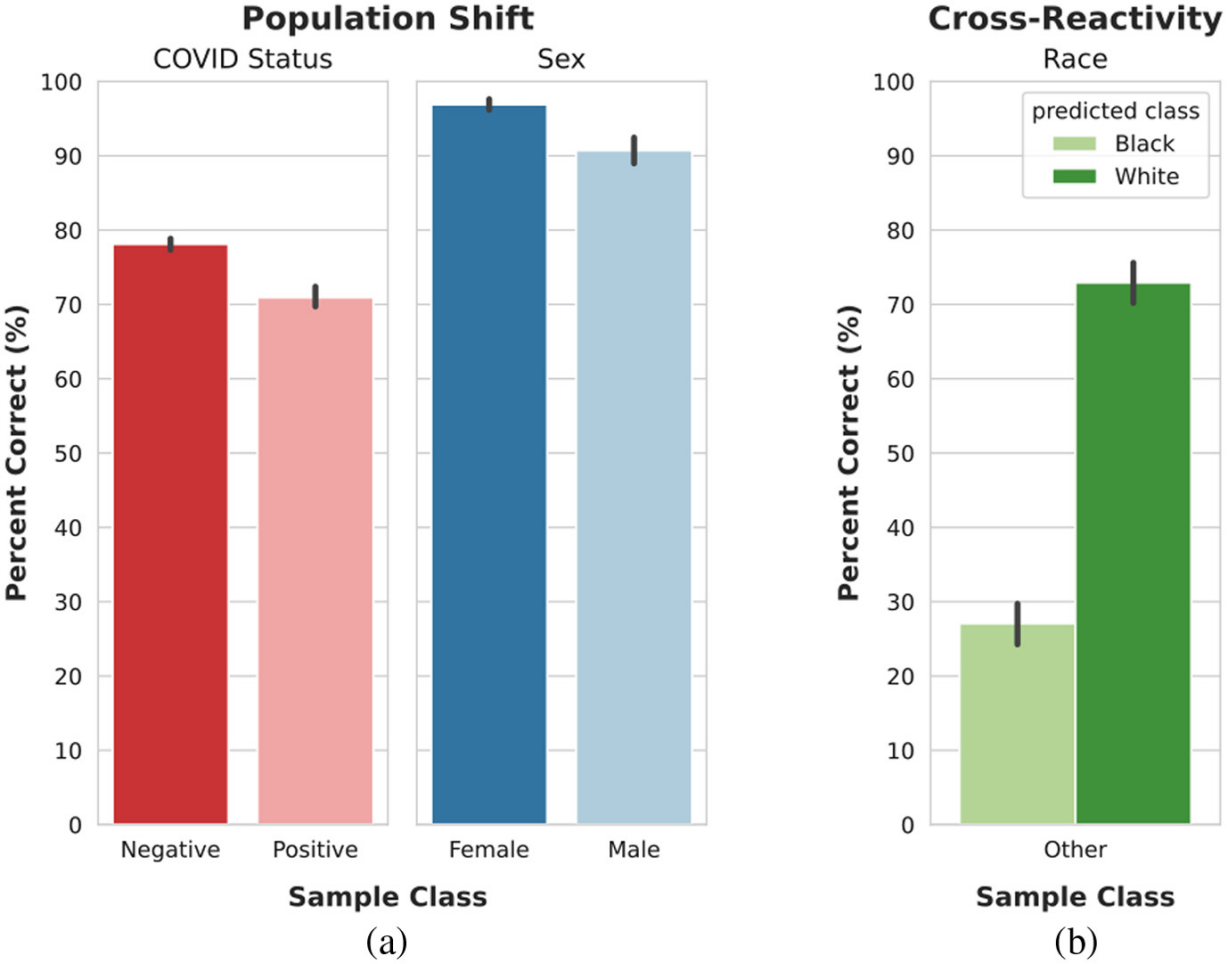
Model classification performance on unrepresented patients (patients whose race was not represented in the training data). (a) Population shift: performance of the sex and COVID status classification tasks. (b) Cross-reactivity: performance of the race classification task.

As previously observed in Sec. 4.1, the decision region analysis indicated the existence of a preferred class for each classification task despite similar performances across classes in the validation-2 original distribution. This trend is also observed in these additional trials, as shown in Fig. 9. Furthermore, the preferred classes (female, white, negative) are the same preferred classes observed in the analysis of the smaller dataset. The model’s behavior in the cases of a population shift and cross-reactivity (Fig. 10) is similar to that of the models trained on a smaller dataset: a tendency to overpredict samples as belonging to the preferred class.

## Data Availability

The Decision Region Analysis for Generalizbility (DRAGen) code is available in a GitHub repository [https://github.com/DIDSR/DRAGen].^33^ The data utilized in this study were obtained from MIDRC (The Medical Imaging and Data Resource Center).

## Disclaimers

The mention of commercial products, their sources, or their use in connection with material reported herein is not to be construed as either an actual or implied endorsement of such products by the Department of Health and Human Services. This is a contribution of the U.S. Food and Drug Administration and is not subject to copyright.
